# COVID-19: Coagulopathy, Risk of Thrombosis, and the Rationale for Anticoagulation

**DOI:** 10.1177/1076029620938149

**Published:** 2020-07-17

**Authors:** Wolfgang Miesbach, Michael Makris

**Affiliations:** 1 Department of Haemostaseology and Hemophilia Center, Medical Clinic 2, Institute of Transfusion Medicine, University Hospital Frankfurt, Germany; 2 Department of Infection, Immunity and Cardiovascular Disease, University of Sheffield, United Kingdom; 3 Sheffield Haemophilia and Thrombosis Centre, Royal Hallamshire Hospital, Sheffield, United Kingdom

**Keywords:** COVID-19, thrombosis, anticoagulation

## Abstract

The novel coronavirus infection (COVID-19) is caused by the new coronavirus SARS-CoV-2 and is characterized by an exaggerated inflammatory response that can lead to severe manifestations such as adult respiratory syndrome, sepsis, coagulopathy, and death in a proportion of patients. Among other factors and direct viral effects, the increase in the vasoconstrictor angiotensin II, the decrease in the vasodilator angiotensin, and the sepsis-induced release of cytokines can trigger a coagulopathy in COVID-19. A coagulopathy has been reported in up to 50% of patients with severe COVID-19 manifestations. An increase in d-dimer is the most significant change in coagulation parameters in severe COVID-19 patients, and progressively increasing values can be used as a prognostic parameter indicating a worse outcome. Limited data suggest a high incidence of deep vein thrombosis and pulmonary embolism in up to 40% of patients, despite the use of a standard dose of low-molecular-weight heparin (LMWH) in most cases. In addition, pulmonary microvascular thrombosis has been reported and may play a role in progressive lung failure. Prophylactic LMWH has been recommended by the International Society on Thrombosis and Haemostasis (ISTH) and the American Society of Hematology (ASH), but the best effective dosage is uncertain. Adapted to the individual risk of thrombosis and the d-dimer value, higher doses can be considered, especially since bleeding events in COVID-19 are rare. Besides the anticoagulant effect of LMWH, nonanticoagulant properties such as the reduction in interleukin 6 release have been shown to improve the complex picture of coagulopathy in patients with COVID-19.

## Introduction

Coronaviruses (CoVs) consist of a large family of single-stranded RNA viruses, identified decades ago but whose clinical significance and epidemic potential were not recognized until the outbreak of severe acute respiratory syndrome CoV (SARS-CoV) and Middle Eastern respiratory syndrome (MERS) in 2002 and 2012, respectively. They can cause symptoms ranging from a mild cold to severe respiratory diseases, with mortality rates of 10% for SARS and 37% for MERS.^1,2^

Severe acute respiratory syndrome CoV 2, the causative agent of COVID-19, is the seventh member of the CoV to be identified and is structurally similar to SARS-CoV, with the 2 viruses sharing about 72% of their genome.^3^ Severe acute respiratory syndrome CoV 2 poses a major threat to global health that goes far beyond the spread and risks of SARS-CoV and MERS. In addition to significant mortality, another key issue of COVID-19 is the exponential increase in the number of infected patients and the very high number of patients in hospitals.

Severe acute respiratory syndrome CoV 2 and other CoVs show similarities and differences. Both viruses can cause fatal lung diseases and appear to be particularly dangerous for elderly people or people with comorbidities.

Like other CoVs, SARS-CoV-2 uses the angiotensin-2 receptor (ACE2) to enter the target cells, but with a higher affinity for ACE2.^3^ After binding to its receptor, ACE2 activates the renin–angiotensin system (RAS), which leads to a downregulation of ACE2 expression, resulting in an increase in angiotensin II (Ang II) and a decrease in its counterpart angiotensin.^1–7^ In contrast to SARS-CoV infection, however, SARS-CoV-2 infection seems less likely to be fatal. The mortality rate varies from country to country and depends on the capacity and performance factors of the health care systems. Despite significantly higher mortality rates for SARS and MERS, COVID-19 has led to more deaths overall due to the high number of infected individuals. By April 16, 2020, more than 2.1 million persons had been infected and more than 142 000 have died of the disease.^5^

While most patients show only mild symptoms,^6^ a characteristic feature of COVID-19 is that a proportion of patients develop severe complications within a short time after infection, such as adult respiratory syndrome (ARDS) or disseminated intravascular coagulation (DIC), sepsis followed by organ failure, and death.^7^ Coagulopathy and thrombotic events have been described in patients with COVID-19, and this review summarizes existing reports and treatment recommendations in patients with CoV infections.

## Coagulopathy and Thrombosis in SARS and MERS

Severe acute respiratory syndrome CoV first appeared 18 years ago.^8^ During the SARS-CoV epidemic in 2002, more than 8000 infected patients and 744 deaths were documented in 26 countries on 5 continents. The main clinical manifestations were upper respiratory symptoms, rapid progression of pneumonia, and about 20% to 30% had to be admitted to intensive care units.^9^ In patients over 65 years of age, the mortality rate was over 50%. Of the patients treated or dying in the intensive care unit, 11.4% developed DIC. In contrast, the mortality rate from sepsis is much lower with an incidence between 75 and 300 per 100 000.^10^

In 2012, a new related zoonotic CoV was identified in the Middle East, the MERS, which causes severe respiratory disease with a mortality rate of over 35%.^11^ Despite the high mortality in both infections, there is no systematic evaluation of the risk of thrombosis and the incidence of thrombosis. There is only 1 case report of pulmonary embolism (PE) in patients with SARS^12^ and 1 case of ischemic stroke.^13^ Other publications reported various clinical and laboratory abnormalities, but no thromboembolic events. A cohort study of 157 SARS patients found no evidence of venous thrombosis or other clotting abnormalities, such as the presence of antibodies to cardiolipin or elevated d-dimer concentrations.^14^ An analysis of laboratory data found that with the exception of thrombocytopenia in 55% of patients and a prolonged activated partial thromboplastin time (aPTT; 40.1-68.1 seconds) in 63%, other coagulation parameters remained unchanged. No elevated d-dimer levels were found except in the 2.5% of the 160 patients who developed DIC. Another study with 138 SARS patients from 2003, 23.2% of whom were admitted to intensive care, showed that the peak d-dimer levels were elevated and platelet count was reduced in patients receiving intensive care or death compared to patients not receiving intensive care.^15^ These laboratory changes are consistent with previous studies which showed that hypoalbuminemia, lymphopenia, and C-reactive protein ≥4 mg/dL were the predictive factors for the progression of pneumonia to respiratory failure in MERS-CoV-infected patients and that elevated lactate dehydrogenase (LDH) levels were associated with hospital-acquired infection with SARS-CoV.^16^

## Coagulopathy and Thrombotic Risk in COVID-19

Similar to SARS and MERS, there is a link between inflammation and severe organ damage in COVID-19 patients. The primary pathology is ARDS, which is characterized by diffuse alveolar damage including hyaline membranes. The viral cytopathic effect of pneumocytes implies direct viral damage.^17^ There is now evidence that some patients may respond to COVID-19 with an exuberant “cytokine storm” response.^18^ Immunological studies have demonstrated that pro-inflammatory cytokines interleukin 6 (IL-6), IL-17A, and tumor necrosis factor α were elevated in the majority of patients with severe outcomes.^19^ Hypercoagulability is an important hallmark of inflammation. Pro-inflammatory cytokines are critically involved in abnormal clot formation and platelet hyperactivation and also play an important role in the downregulation of important physiological anticoagulant pathways.^20^

Other patient-related, pneumonia-related, and SARS-CoV2-related factors can lead to a significantly higher risk of thrombotic complications in patients with COVID-19 (Table 1). In COVID-19, risk factors for the development of severe symptoms are advanced age, male sex, and presence of comorbidities, especially in hypertension where a hazard ratio (HR) of 1.70 to 3.05 for death has been demonstrated.^21,22^ In general, hypertension was identified as an independent risk factor for deep vein thrombosis in a large prospective study involving more than 18 000 patients.^23^ Elderly patients and those with comorbidities are more likely to develop severe complications of COVID-19 infection and have a higher risk of thrombosis.^24–26^

**Table 1.** Factors Increasing the Risk of Thrombosis.

Patient related	Pneumonia related	SARS-CoV-2 related
Age	ICU	Angiotensin ↑
Male sex	Central vein catheters	Cytokines ↑
Hypertension	Endothelial damage and increase of FVIII/VWF	Tissue factor ↑
Cardiovascular morbidity	Increase of HIF-1	PAI-1↑
Immobilization		

Abbreviations: HIF-1, hypoxia-inducible factor; ICU, intensive care unit; PAI, plasminogen activator inhibitor-1; SARS-CoV-2, severe acute respiratory syndrome corona virus 2; VWF, von Willebrand factor.

The RAS plays an important role in COVID-19, with angiotensin-converting enzyme 2 (ACE2) acting as a functional SARS-CoV-2 receptor, resulting in the downregulation of ACE2 and higher expression of Ang II.^27,28^ Angiotensin-converting enzyme 2 is predominantly expressed by the vascular endothelial cells of the lung, but also in extrapulmonary tissue, heart, nervous system, intestine, kidneys, blood vessels, and muscles on cell surfaces, which may explain the multi-organ dysfunction observed in patients with COVID-19.^29^ Angiotensin II is known to be one of the most potent vasoconstrictors^30^ and also increases hypercoagulability by increasing expression of tissue factor^31^ and plasminogen activator inhibitor 1. Markedly elevated Ang II levels have been reported in patients with COVID-19.^32^

Coagulopathy has been described in studies that document clinical and laboratory changes in COVID-19 patients in up to 50% of those with severe manifestations.^22^ Several studies confirm the relevance of elevated d-dimer concentrations in COVID-19. Besides the known variability in healthy volunteers and their tendency to increase with age, there is an association of elevated d-dimer levels and fibrin degradation products under all conditions with an activated coagulation system, such as thrombosis, infection, or malignancy.^33^

The increase in d-dimer was the most significant change in coagulation parameters in COVID-19 patients and occurred more frequently than other coagulation parameters such as prothrombin time (PT) or aPTT.^34^ Furthermore, the coagulation parameters indicate a marked tendency to thrombosis, as the changes of other parameters indicating a bleeding tendency, such as severely low platelets or fibrinogen levels, were absent. Comparing the result from severely to nonseverely affected patients, the median fibrinogen levels, with the exception of 28.6% where the fibrinogen levels were below 1 g/L, increased to supraphysiological values (5.16 g/L, interquartile range [IQR]: 3.74-5.69 g/L), and the antithrombin levels remained in the normal range. Remarkably, increased d-dimers measured at hospital admission could predict a severe outcome for COVID-19. In a multivariate analysis comparing clinical and laboratory parameters of 137 survivors to 54 nonsurvivors, the mortality odds ratio [OR] for d-dimer levels > 0.5 µg/mL was 2.14, for d-dimer levels > 1 µg/mL was 18.42, while for a PT >16 seconds,^22^ the OR was 4.62. The death of the patients occurred at a median of 18 days of hospital treatment after the patients were mechanically ventilated for 14.5 days. All 54 deceased patients developed sepsis (100% vs 42% of survivors), 53 patients suffered from respiratory failure (98% vs 36%), 50 patients suffered from acute respiratory failure (ARDS; 93% vs. 7%), 28 patients suffered from heart failure (52% vs 12%), and 38 patients suffered from septic shock (70% vs 0%). The deceased patients were significantly older (64 ± 20.7 years vs 52.4 ± 15.6 years), significantly more male, and with chronic diseases (57.1% vs 38.9%). In summary, coagulopathy occurred in 50% of patients who died versus in 7% of patients who survived. Other serious complications included acute heart damage (59% vs 1%), acute kidney damage (50% vs 1%), and secondary infections (50% vs 1%). In patients who died of COVID-19, the sepsis-related organ failure assessment (SOFA) score was 4.5 points at admission, while the survivors’ score was only 1.0.

A large study that included 1099 COVID-positive patients from 552 hospitals in China found that d-dimer concentrations above the threshold of 0.5 mg/L were observed in 46.4% of the patients; 60% of them developed severe manifestations. In these patients, d-dimer levels of 2.12 µg/mL (0.77-5.27) were 4 times higher^26^ compared to nonseverely affected patients (0.61 µg/mL, 0.35-1.29). Thus, d-dimer concentration and SOFA score provide, in addition to the age of the patient, important information on the prognosis of COVID-19 disease. Other risk factors for a severe outcome were lymphopenia, leukocytosis, and increased laboratory values of alanine aminotransferase, LDH, highly sensitive troponin I, creatine kinase, serum ferritin, IL-6, PT, creatinine, and procalcitonin.^24^

## Incidence of Thrombosis in COVID-19 Patients

Laboratory and imaging studies found an increased risk of thrombotic complications in patients with COVID infection. The precise incidence of thrombosis in patients with COVID-19 has not been determined. There are large studies from China that investigate the clinical course of COVID-19 patients. However, there was no evidence for the presence of thrombosis, PE, or arterial thrombotic complications: A summary of a report on 72 314 COVID-19 cases from the Chinese Center for Disease Control and Prevention mentioned only the age range of the affected patients with 14.8% in patients aged ≥80 and that a critical course occurred in 5% of patients with a mortality rate of 49%.^35^ In a study of 1099 COVID-positive patients from 552 hospitals in China, the high frequency of elevated d-dimer values in 46.4% of patients was highlighted, without, however, further investigating its influence on the thrombotic risk.^26^

When patients were specifically screened for the presence of thrombosis, a remarkably high rate was identified (Table 2). These studies included patients with varying degrees of disease severity. In a retrospective evaluation of 138 patients, both the thrombotic risk (using the Padua prediction score) and the bleeding risk were assessed. A total of 16.67% of mostly critically ill patients with a high risk for thrombotic events were identified, of which 17.3% were diagnosed with deep vein thrombosis^36^ despite the use of guideline-recommended thromboprophylaxis. Deep vein thrombosis was diagnosed by ultrasound 3 to 18 days after hospital admission. The most frequent Padua risk parameters were acute infections (100%), heart or respiratory failure (39.9%), limited mobility (15.2%), and age (12.3%). These criteria continued to increase in critically ill patients and led to significantly higher score values.^36^

**Table 2.** Incidence of Thrombotic Events.

Study	Patients (n)	Severity of disease	Thrombotic event	Use of LMWH
Xu et al^36^	23	Critically ill and high risk of thrombosis	DVT in 17,3%	Yes
Chen et al^37^	25	Proven COVID-19 pneumonia	PE in 40%	Yes in 80%
Cui et al^38^	81	Severe and nonsevere	DVT in 25%	No
Klok et al^39^	184	Proven COVID-19 pneumonia	31% (81% PE)	Yes
Zhang et al^40^	241	Critically ill	Stroke in 5.7%	ND

Abbreviations: DVT, deep vein thrombosis; LMWH, low-molecular-weight heparin; ND, not determined; PE, pulmonary embolism.

Patients with COVID-19 pneumonia also have a high risk of PE, and a rising d-dimer value facilitates the diagnosis of a thromboembolic event. Out of 1008 hospitalized patients, 25 with confirmed pneumonia underwent computed tomography pulmonary angiography.^37^ Pulmonary embolism was diagnosed in 40% mainly localized in small branches of the pulmonary artery. Interestingly, these patients showed significantly higher median d-dimer levels (11.07 µg/mL; IQR: 7.12-21.66) compared to median d-dimer levels of 2.44 µg/mL (IQR: 1.68-8.34) in patients without PE.

In a single-center retrospective study, 81 patients with COVID-19 were described who had to be admitted to an intensive care unit and the incidence of deep vein thrombosis was determined. The patients did not receive thromboprophylaxis. Twenty patients (25%) had DVT of the lower extremities, 40% of whom died. The incidence of PE was not systematically investigated. The use of a d-dimer cutoff >1.5 µg/mL to predict DVT showed a sensitivity of 85% and a specificity of 88.5%.^38^

The occurrence of thrombosis has also been reported in patients using prophylactic low-molecular-weight heparin (LMWH). A study of 184 patients with COVID-19 pneumonia from 3 Dutch hospitals investigated the incidence of symptomatic acute PE, DVT, ischemic stroke, myocardial infarction, or systemic arterial embolism in COVID-19 patients admitted to intensive care.^39^ There was a 31% incidence of thrombosis. All patients received at least standard doses for LMWH thromboprophylaxis, although schedules differed between hospitals and doses increased over time. In 9.2%, therapeutic anticoagulation was administered on admission. It is remarkable that none of the patients developed DIC. The majority of patients suffered from PE (81%), but a thrombotic stroke occurred in 3 patients.^39^ Coagulopathy and changes in global coagulation markers and spontaneous prolongation of PTT and PT were independent predictors of thrombotic complications (PT >3 seconds and aPTT >5 seconds; adjusted HR: 4.1; 95% CI: 1.9-9.1).

Arterial thrombosis has also been reported. Of 241 patients, 5.7% suffered from acute cerebrovascular disease,^40^ 1 patient additionally from of ischemia in the lower limbs bilaterally as well as in two fingers. In these patients, an unusual combination of antiphospholipid antibodies with the presence of IgA anticardiolipin and anti-β2 glycoprotein I IgA and IgG antibodies was detected raising the question of the role of antiphospholipid antibodies. However, these were antibodies detected on a single occasion and no titers were given, so by definition did not satisfy the criteria for antiphospholipid syndrome.

It was not clear from the clinical studies whether the thrombotic pulmonary complications were PE or primary pulmonary thrombosis, as only a small number of deep vein thromboses were detected in these patients.

The results of autopsy studies indicate the presence of pulmonary endothelial damage and microthrombosis. In a case series of 4 autopsies of COVID-19-infected patients from New Orleans with sudden respiratory decompensation, it was shown that there were no thromboembolisms in the major pulmonary arteries, but small thrombi were present in sections of the peripheral lung parenchyma.^41^ Furthermore, the microscopic findings confirmed that small vessels contained thromboembolisms and small thrombi together with scattered areas of diffuse alveolar damage, indicating that small vessels can a be affected by microthrombosis. d-dimers determined near the time of death were elevated in only 2 of the 4 patients.

In another case series of 27 autopsies, SARS-CoV-2 was detected in the endothelial cells of several organs, with the highest concentrations found in the respiratory tract and lower concentrations in the kidneys, liver, heart, brain, and blood.^42^ Therefore, tissues beyond the respiratory tract may be affected, which may contribute to the clinical course of COVID-19 and possibly exacerbate preexisting conditions. This suggests that SARS-CoV-2 may lead to a generalized inflammatory response of the endothelium, causing fatal organ failure.

A further postmortem analysis found diffuse endothelial inflammation,^43^ which may explain why many COVID-19 patients die not only from pneumonia but also from multiple organ failure due to severe microcirculation disorders, activation of complement pathways, and a related procoagulant state. Patients with preexisting endothelial dysfunction (high blood pressure, cardiovascular disease, or diabetes) are therefore more likely to be affected.

## The Rationale for Individualized Use of Anticoagulation in COVID-19

The clinical spectrum of infection with the novel SARS-CoV-2 ranges from the absence of any symptoms to fatal septic shock. The transition from mild to severe in patients with COVID-19 may be caused by cytokine storms and increased hypercoagulability. As with all coagulopathies, treatment of the underlying disease is mandatory.

For COVID-19, it is advisable to offer prophylactic anticoagulation with LMWH as early as possible to prevent thrombotic events and organ damage. This was recommended in the recently published preliminary International Society on Thrombosis and Haemostasis (ISTH) guidance on the detection and treatment of coagulopathy in COVID-19.^44^ The ISTH guidance document provides risk stratification on the admission of COVID-19 patients and treatment of a potentially developing coagulopathy. It suggests that patients with an elevated d-dimer (ie, arbitrarily defined as a 3- to 4-fold increase) should be admitted to hospital. Low-molecular-weight heparin should be considered in all patients who need to be hospitalized for COVID-19 infection unless contraindicated.

Bleeding can be caused by DIC and sepsis, which are common in severe cases. In a study by Tang et al from Wuhan, 71% of nonsurvivors of COVID-19 infection met the ISTH criteria for DIC compared to 0.4% of survivors.^34^ However, a significant reduction in other clotting parameters such as platelets or antithrombin, the most commonly associated with DIC, has not been described and no bleeding events have been reported even in severe cases of COVID-19.

In a study of 449 patients with severe COVID-19 manifestations, 99 of them received heparin (mainly LMWH) for 7 days or longer.^45^ The 28-day mortality between heparin users and nonusers was compared; comparison was also made with regard to the different risk of coagulopathy stratified by the sepsis-induced coagulopathy score (SIC) and d-dimer value. d-dimer, PT, and age were positively and platelet count was negatively correlated with 28-day mortality in multivariate analysis. No difference in 28-day mortality was found between heparin users and nonusers (30.3% vs 29.7%, *P* = .910). However, the 28-day mortality of heparin users was lower than that of nonusers in patients with SIC score ≥4 (40.0% vs 64.2%, *P* = .029) or d-dimer >6 times the upper limit of normal value (32.8% vs 52.4%, *P* = .017).

Several nonanticoagulant properties of LMWH have also been suggested, such as the reduction of the release and biological activity of IL-6.^46,47^ Low-molecular-weight heparin has been shown to bind to SARS-CoV-1 and block replication of the virus.^48^ Recently, the anti-inflammatory effect of LMWH was confirmed in patients with COVID-19 with lower IL-6 levels and higher lymphocyte levels compared to COVID-19 patients not treated with LMWH, while changes in other inflammatory factors were not statistically significant.^49^ In addition, initially elevated levels of d-dimer and fibrinogen degradation products decreased significantly after treatment with LMWH, indicating an improvement in hypercoagulable state in COVID-19 patients. The clinical benefit of LMWH may be due to its effect on inhibiting the release of IL-6 together with the increase in lymphocytes that can delay or block the inflammatory cytokine storm.

Although the concept of using LMWH in any hospitalized COVID-19 patient is generally accepted, there is a debate about the dosage to be employed. Since there are reports that thrombosis occurred despite low-dose prophylactic use of LMWH, dose escalation of LMWH can be employed either empirically or based on increasing d-dimer values. Ideally, we should wait for the randomized clinical trials to report first, but the impact of the disease now means that decisions may be made empirically and are largely based on opinion.

However, the most reliable approach to evaluating the effects of new drugs is randomized clinical trials. Even in the event of epidemics or pandemics and the urgent need for rapid and effective treatment, randomized trials should be conducted as early as possible. In uncontrolled trials, several multiple drugs could be selected without testing a clear risk–benefit ratio in the typically variable clinical courses of new diseases.^50^

## Antithrombotic Treatment in COVID-19 Patients

Therapeutic anticoagulation is the cornerstone for the treatment of thrombosis and PE. For the treatment of venous thromboembolism in intensive care units, unfractionated heparin is typically preferred because of its short mode of action and no known interaction with any of the investigational drugs of COVID-19.^51^ However, frequent monitoring is required, and monitoring with aPTT may be compromised by increased activity of the acute phase protein FVIII. In this case, the aXa test should be preferred.

Longer acting agents, such as LMWH, may also be considered. It can be administered subcutaneously once or twice daily and does not require frequent monitoring to ensure that it is effectively dosed.

Oral anticoagulants, including warfarin, the direct thrombin inhibitor dabigatran, and the factor Xa inhibitors apixaban, rivaroxaban, edoxaban, and betrixaban, should not be considered for the treatment of thrombosis in COVID-19 patients, inter alia, because of possible interactions with antiviral therapeutics.^51^

In 3 severe cases of COVID-19-related ARDS, intravenous administration of recombinant tissue plasminogen activator was used. The authors reported a temporary improvement in respiratory failure even in the absence of manifest PE, suggesting the contribution of lung microthrombi in the prothrombotic state of COVID-19.^52^

Finally, autopsy findings of endothelial cell infections, endotheliitis, and microthrombosis may lead to an additional, more targeted treatment with endothelial stabilizing drugs, such as anti-inflammatory drugs, anticytokines, ACE inhibitors, and statins.

## Summary

There is a growing understanding of the pattern of COVID-19 not only in epidemiology and immunology but also in subsequent coagulopathy and coagulopathy treatment strategies. COVID-19 is associated with a hypercoagulable state, and infected patients with additional risk factors have a worse outcome. Initial data suggest high thromboembolism rates in patients without and often with standard pharmacological thromboprophylaxis. Limited data also suggest that localized pulmonary microvascular thrombosis may play a role in the progressive respiratory failure. Most evidence is limited by small retrospective studies, and the true prevalence of thrombosis in COVID-19 still needs to be evaluated in larger studies.

Severe complications of COVID-19 occur more frequently in older patients with comorbidities, and this group of individuals also have an age-related increased risk of thrombosis. The high risk of thrombosis in COVID-19 is demonstrated by the increase in d-dimer, which was found to be the most significant change in coagulation parameters in COVID-19 patients, suggesting increased thrombin production and activation of fibrinolysis.

Despite the fact that d-dimer is a nonspecific acute phase reactant and may be elevated by other causes and inflammation, it has been shown that a consecutive increase in d-dimer may indicate the presence of thrombosis and PE in critically ill COVID-19 patients. A significant reduction in other, coagulation parameters such as platelets, fibrinogen, or antithrombin, which are most frequently associated with DIC, has not been frequently described, neither have bleeding events during COVID-19. There is therefore a need to identify the increased risk of thrombotic events at an early stage and to prevent thrombotic events and organ damage as far as possible. Prophylactic anticoagulation with LMWH should be initiated as soon as possible to prevent thrombotic events and counteract the pro-inflammatory influence of cytokines and other factors.

The incidence of thrombosis in critically ill patients is high and thrombotic events occurred despite the prophylactic use of LMWH. There is an urgent need for the results from randomized trials regarding the appropriate antithrombotic prophylaxis and treatment. Risk stratification according to d-dimer values may be an option to individualize treatment or to use higher doses even taking into account the complex thrombotic picture of COVID-19.
